# Constraints on the Ecomorphological Convergence of Zooplanktivorous Butterflyfishes

**DOI:** 10.1093/iob/obab014

**Published:** 2021-05-13

**Authors:** J R Hodge, Y Song, M A Wightman, A Milkey, B Tran, A Štajner, A S Roberts, C R Hemingson, P C Wainwright, S A Price

**Affiliations:** 1 Department of Biological Sciences, Clemson University, Clemson, SC 29634, USA; 2 Department of Evolution and Ecology, University of California, Davis, Davis, CA 95616, USA; 3 Department of Biomedical Engineering, The Chinese University of Hong Kong, Hong Kong; 4 Harbor Branch Oceanographic Institute, Florida Atlantic University, Fort Pierce, FL 34946, USA; 5 College of Science and Engineering, James Cook University, Townsville, QLD 4811, Australia

## Abstract

Whether distantly related organisms evolve similar strategies to meet the demands of a shared ecological niche depends on their evolutionary history and the nature of form–function relationships. In fishes, the visual identification and consumption of microscopic zooplankters, selective zooplanktivory, is a distinct type of foraging often associated with a suite of morphological specializations. Previous work has identified inconsistencies in the trajectory and magnitude of morphological change following transitions to selective zooplanktivory, alluding to the diversity and importance of ancestral effects. Here we investigate whether transitions to selective zooplanktivory have influenced the morphological evolution of marine butterflyfishes (family Chaetodontidae), a group of small-prey specialists well known for several types of high-precision benthivory. Using Bayesian ancestral state estimation, we inferred the recent evolution of zooplanktivory among benthivorous ancestors that hunted small invertebrates and browsed by picking or scraping coral polyps. Traits related to the capture of prey appear to be functionally versatile, with little morphological distinction between species with benthivorous and planktivorous foraging modes. In contrast, multiple traits related to prey detection or swimming performance are evolving toward novel, zooplanktivore-specific optima. Despite a relatively short evolutionary history, general morphological indistinctiveness, and evidence of constraint on the evolution of body size, convergent evolution has closed a near significant amount of the morphological distance between zooplanktivorous species. Overall, our findings describe the extent to which the functional demands associated with selective zooplanktivory have led to generalizable morphological features among butterflyfishes and highlight the importance of ancestral effects in shaping patterns of morphological convergence.

## (Portuguese abstract) Resumo

A evolução de estratégias similares para suprir as demandas de nichos ecológicos compartilhados em organismos pouco relacionados, depende da sua história evolutiva e da natureza das relações entre forma e função. Em peixes, a identificação visual e o consumo de zooplanctôn microscópico, a zooplanctivoria seletiva, é um tipo distinto de forrageamento frequentemente associado a um conjunto de especializações morfológicas. Estudos anteriores identificaram inconsistências na trajetória e magnitude das mudanças morfológicas que surgem a partir das transições para a zooplanctivoria seletiva, fazendo alusão à diversidade e importância dos efeitos ancestrais. Aqui investigamos se transições para a zooplanctivoria seletiva influenciaram a evolução morfológica dos peixes-borboleta marinhos (família Chaetodontidae), um grupo especialista em presas pequenas conhecido pelos muitos tipos de bentivoria de alta precisão. Utilizando uma estimativa ancestral bayesiana, inferimos a evolução recente da zooplanctivoria dentre os ancestrais bentívoros que caçavam pequenos invertebrados e alimentavam-se de pólipos de coral. Características relacionadas a captura de presa parecem ser funcionalmente versáteis com pouca distinção entre as espécies com modo de forrageamento bentívoro e planctívoro. Em contraste, várias características relacionadas a detecção da presa ou capacidade natatória estão evoluindo em direção a um novo ótimo, específico para a zooplanctivoria. Apesar da história evolutiva relativamente recente, uma morfologia geral comum, e evidência de uma restrição na evolução do tamanho corporal, a evolução convergente reduziu significativamente a distância morfológica entre as espécies zooplanctívoras. No geral, nossos resultados descrevem até que ponto as demandas funcionais associadas à zooplanctivoria seletiva levaram a características morfológicas generalizadas nos peixes-borboleta e destacam a importância dos efeitos ancestrais em moldar os padrões de convergência morfológica.

## (Spanish abstract) Resumen

El hecho de que organismos con parentesco lejano evolucionen estrategias similares para satisfacer las demandas de un nicho ecológico compartido depende de su historia evolutiva y de la naturaleza de la relación forma-función. En peces, la identificación visual y el consumo de plancton microscópico, la zooplanctivoría selectiva, es un tipo específico de alimentación usualmente asociado a un conjunto de especializaciones morfológicas. Estudios previos han identificado inconsistencias en la trayectoria y magnitud de cambios morfológicos tras transiciones hacia zooplanctivoría selectiva, aludiendo a la diversidad e importancia de efectos ancestrales. Aquí investigamos si las transiciones a zooplanctivoría selectiva han influido en la evolución morfológica de los peces mariposa marinos (familia Chaetodontidae), un grupo especializado en presas pequeñas conocido por varios tipos de alimentación de alta precisión en el bentos. Usando una estimación de estado ancestral Bayesiana, inferimos la evolución reciente de la zooplanctivoría entre ancestros bentívoros que cazaron pequeños invertebrados y se alimentaron de pólipos de coral. Los rasgos relacionados con la captura de presas parecen ser versátiles funcionalmente con escasa distinción morfológica entre especies con modos de alimentación bentívoros y planctívoros. En cambio, múltiples rasgos relacionados con la detección de presas o con la capacidad natatoria están evolucionando hacia un nuevo óptimo específico para zooplanctivoría. A pesar de una historia evolutiva relativamente corta, una morfología general común, y evidencia de restricción en la evolución del tamaño de los peces, una evolución convergente ha reducido la distancia morfológica entre especies zooplanctívoras de forma casi significativa. En conclusión, nuestros hallazgos describen hasta qué punto las demandas funcionales asociadas con la zooplanctivoría selectiva han desembocado en rasgos morfológicos generalizados en peces mariposa y destacan la importancia de los efectos ancestrales en la creación de patrones de morfología convergente.

## Introduction

All organisms are tasked with the functional challenge of acquiring energy, and those that target the same resources often converge on similar strategies or morphological features. Many intrinsic and extrinsic factors can influence contemporary ecomorphological associations, including evolutionary history and the nature of form–function relationships (Conway Morris 2008; Stayton 2008; Losos 2011). For example, ancestral differences in phenotype, genetic variation, or internal constraints may cause divergence toward distinct solutions optimized to meet the same functional challenge (Arnold 1994; Donoghue 2005; Losos 2010). Alternatively, the same solution may be achieved through functional rather than phenotypic convergence (Wainwright 2007; Young et al. 2010). Moreover, functionally versatile morphologies capable of exploiting diverse resources can confound convergent patterns if morphological features that confer high fitness in a particular selective environment evolved initially for another reason—referred to as exaptation (Losos 2011; Zelditch et al. 2017). Phylogenetic comparative methods are powerful tools that can be used to infer the causative processes of ecomorphological patterns, as well as quantify the role of ancestral effects and establish a foundation for further investigation of form–function relationships (Maynard Smith et al. 1985; Harvey and Purvis 1991; Losos 2011; Moen et al. 2016).

In fishes, the relationship between resource use and morphology has been widely studied. Modifications of form corresponding with transitions to herbivory (Davis and Betancur-R 2017), ectoparastivory (Baliga and Mehta 2018), insectivory (Pos et al. 2019), durophagy (Collar et al. 2014), piscivory (Collar et al. 2009), and zooplanktivory (Cooper et al. 2017), among others, have been identified. Zooplanktivory is a particularly distinct trophic niche, commonly associated with unique selective pressures thought to influence the diversification of traits related to swimming performance and the capture and processing of small prey (Davis and Birdsong 1973; Lazzaro 1987; Hobson 1991). Fishes that feed on zooplankton generally employ either a filtering strategy, whereby they swim with their mouths open through the water and passively engulf planktonic prey, or a selective strategy, whereby drifting zooplankters are visually targeted and consumed via suction feeding (Lazzaro 1987). Selective zooplanktivory is the more common strategy among acanthopterygians (Hobson 1991). Functionally, selective zooplanktivory poses specific sensory, feeding, and locomotory challenges, including the detection and capture of small (<3 mm), typically transparent and highly evasive prey as they drift through the water column (Lazzaro 1987). Despite these seemingly uniform functional demands and the repeated evolution of selective zooplanktivory, the direction and magnitude of associated morphological change is varied and there are several examples of incomplete convergence (Wainwright et al. 2002, 2004; Cooper and Westneat 2009; Aguilar-Medrano et al. 2011; Schmitz and Wainwright 2011; Frédérich et al. 2013; Friedman et al. 2016; Cooper et al. 2017; Tavera et al. 2018).

**Fig. 1:**
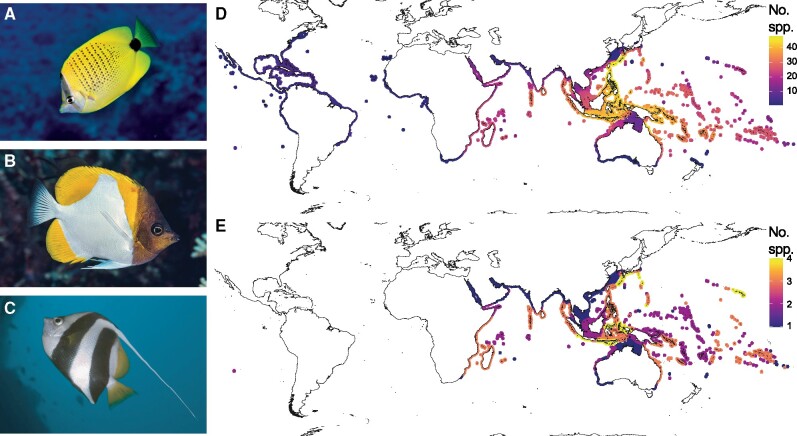
Examples of butterflyfish whose primary mode of foraging is selective zooplanktivory include (**A**) *Chaetodon miliaris*, (**B**) *Hemitaurichthys polylepis*, and (**C**) *H. diphreutes*. Photographs with permission from (A) Luiz Rocha, (B) François Libert, and (C) Sven De Vos. (**D**) Butterflyfishes are associated with reefs throughout the world’s tropical and sub-tropical oceans, with high species richness in the IAA that decreases outwardly. The heatmap shows the alpha diversity distribution of 128 butterflyfish species with spatial data available from the IUCN Red List spatial database (IUCN 2020). See Supplementary Table S1 for information on the species included. (**E**) Zooplanktivorous butterflyfish species are restricted to the Indo-Pacific and have high alpha diversity in areas within and peripheral to the IAA. The heatmap shows the alpha diversity distribution of 11 zooplanktivorous species with spatial data available from the IUCN Red List spatial database (IUCN 2020). Both maps use the WGS 84 projection.

The functional challenges, selective pressures, and predicted morphological features of selective zooplanktivores (Table 1) are typically contrasted with those of general piscivores and benthic foragers, with the presumption that ancestral lineages fed on comparatively large prey, and, through evolutionary time, trended toward feeding on smaller prey (Gosline 1971; Davis and Birdsong 1973; Lazzaro 1987; Kotrschal 1988; Hobson 1991). The trophic morphology of selective zooplanktivores is predicted to facilitate suction feeding on prey presumed to be smaller than those consumed by their ancestors (Davis and Birdsong 1973; Lazzaro 1987; Hobson 1991); while locomotor traits are predicted to increase swimming efficiency and speed in mid-water (Davis and Birdsong 1973; Hobson and Chess 1978; Hobson 1991). Indeed, there is evidence of convergence toward some of these morphological optima within certain groups of marine teleosts, including the Labridae (Wainwright et al. 2002, 2004; Schmitz and Wainwright 2011), Pomacentridae (Cooper and Westneat 2009; Aguilar-Medrano et al. 2011; Frédérich et al. 2013; Cooper et al. 2017), Acanthuridae (Friedman et al. 2016), and Haemulidae (Tavera et al. 2018), and a general reduction in the size of morphological features related to feeding following transitions to selective zooplanktivory (Schmitz and Wainwright 2011; Frédérich et al. 2013; Friedman et al. 2016). However, selective zooplanktivory has evolved independently in many of the major coral reef fish families, along disparate lineages that have ancestrally occupied a variety of trophic niches (Hobson 1991; Wainwright and Bellwood 2002). Differences in ancestral condition and the mapping of form to function amongst these lineages likely accounts for some of the inconsistencies in the trajectory and magnitude of morphological changes identified thus far (Wainwright and Bellwood 2002). Yet, few ecomorphological studies have investigated convergence at scales sufficient to assess the contribution of ancestral effects following transitions to selective zooplanktivory. Butterflyfishes (family Chaetodontidae) provide an ideal opportunity to investigate this, as they comprise species with several different forms of benthivory, as well as selective zooplanktivory (Fig. 1A–C).

**Table 1:** Predicted morphological modifications of selective zooplanktivores relative to general piscivores and benthivores, and the associated functional challenges and selective pressures thought to influence their evolution

	Predicted modifications	Reasoning (References)
Feeding traits		
Eyes	Large	Improves visual acuity, aiding in the detection and capture of small, typically transparent, and highly evasive prey against a low-contrast background (Jones 1968; Davis and Birdsong 1973; Lazzaro 1987; Fernald 1990; Wainwright and Richard 1995; Johnsen 2001; Wainwright and Bellwood 2002).
Ascending premaxillary process	Long	Increases jaw protrusion, ram velocity (Westneat and Wainwright 1989; Ferry-Graham et al. 2001; Waltzek and Wainwright 2003), the acceleration of fluid around the prey (Holzman et al. 2008; Staab et al. 2012), and potentially suction strength (Motta 1982, 1984b; Lazzaro 1987; Wainwright and Richard 1995; Wainwright and Bellwood 2002; Cooper and Westneat 2009).
Mouth	Small	Produces a steeper pressure gradient that exerts a greater force on the prey, thereby increasing suction strength (Jones 1968; Davis and Birdsong 1973; Motta 1982, 1988; Hobson 1991; Wainwright and Richard 1995; Wainwright and Bellwood 2002; Wainwright et al. 2007).
Epaxial musculature	Large	Produces stronger forces to actuate the kinematic events that generate buccal pressure gradients, potentially increasing suction strength by Motta (1982) and Carroll et al. (2004).
Dentition	Reduced	Reduced function in prey capture and processing (Davis and Birdsong 1973; Lazzaro 1987; Motta 1988; Hobson 1991).
Gill rakers	Long, numerous, finely-toothed	Beneficial for retaining small prey items (Davis and Birdsong 1973; Lazzaro 1987).
Locomotor traits		
Body shape	Fusiform, streamlined	Reduces drag, thereby increasing mid-water swimming efficiency and speed (Lighthill 1970; Davis and Birdsong 1973; Hobson and Chess 1978; Webb 1984a; Weihs 1989; Hobson 1991).
Caudal fin shape	Semi-lunate, lunate, deeply forked	Increases thrust and mid-water swimming efficiency and speed (Davis and Birdsong 1973; Hobson and Chess 1978; Webb 1984b; Weihs 1989; Hobson 1991).
Body size	Large or	Increases swimming speed, which is beneficial for avoiding predation (Hobson 1991).
	Small	Accommodates small prey capture (Davis and Birdsong 1973; Cohen et al. 1993).

Feeding traits function in prey detection and capture, while locomotor traits influence swimming performance.

**Fig. 2:**
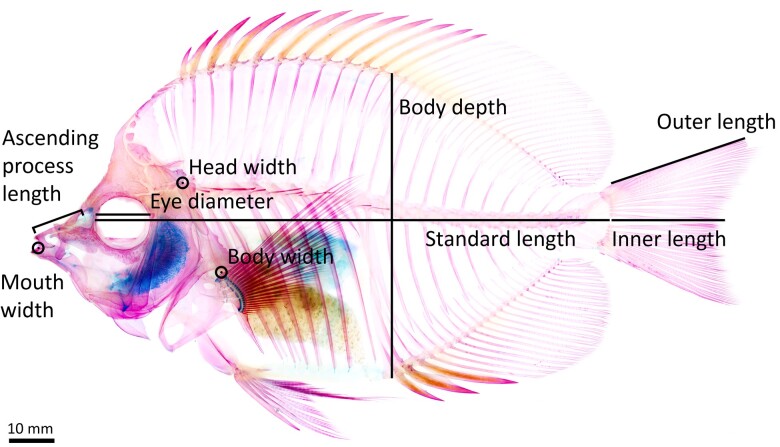
Lateral view of a cleared and stained zooplanktivorous butterflyfish (*H. thompsoni*), illustrating the linear morphometric measurements. Circled points indicate the location of width measurements.

Butterflyfishes are among the most conspicuous inhabitants of coral reefs around the world. Their distribution mirrors that of reef-associated fishes generally, with high species richness in the Indo-Australian Archipelago (IAA) that decreases outwardly (Fig. 1D; Bellwood et al. 2005; Allen 2008), and areas of high endemism in the Red Sea, Hawaii, and Easter Island (Hodge et al. 2014; Kulbicki et al. 2014). Butterflyfishes are well known for their high-precision benthic foraging (Goatley and Bellwood 2009; Goatley et al. 2010), which includes browsing and grazing obligately or facultatively on corals, and hunting benthic invertebrates (Motta 1988; Cole and Pratchett 2013). Eleven of the 134 nominal species are known to be selective zooplanktivores (∼8% of the family) that primarily consume larvaceans and highly evasive calanoid copepods (Fig. 1A–C and E and Supplementary Table S1; Hobson 1974; Harmelin-Vivien and Bouchon-Navaro 1983; Sano 1989). Because transitions to selective zooplanktivory can require shifts in both prey type and foraging habitat, the associated selective pressures may impact feeding and locomotor morphology independently. Contrasting patterns of cranial and post-cranial evolution have been identified in butterflyfishes (Konow et al. 2017), and more broadly in marine teleosts (Friedman et al. 2020). Accordingly, we selected morphological traits based on previous descriptions of their functional relevance to prey detection and capture (feeding traits) and swimming performance (locomotor traits; Fig. 2 and Table 1).

Transitions in trophic niche are frequently accompanied by changes in feeding morphology (Liem 1980; Christiansen and Wroe 2007; Cooper et al. 2017), and previous work has described the feeding functional morphology of several butterflyfish species, including specializations of the zooplanktivore *Chaetodon miliaris* (Motta 1982, 1984a, 1985, 1987, 1988, 1989). However, all butterflyfishes specialize on small prey and their small, moderately protrusible, and laterally enclosed mouths have been proposed as adaptations toward improved biting and thus benthivory (Kotrschal 1988) and, conversely, likened to those of other specialized planktivores (Coughlin and Strickler 1990; Hobson 1991). Moreover, others have argued that zooplanktivorous butterflyfishes are indistinguishable from their benthivorous congeners on the basis of external morphology (Hobson 1991). Indeed, their deep-bodied morphology is highly conserved across species (Hodge et al. 2018). Therefore, butterflyfish trophic morphology may be exapted to selective zooplanktivory, masking any pattern of ecomorphological convergence particularly in conjunction with a conserved external body shape. Here we assess whether zooplanktivorous butterflyfishes express convergent morphologies that are distinct from their benthivorous congeners.

## Materials and methods

### Foraging and morphometric data collection and handling

We used data on dietary habits (Cole and Pratchett 2013) to categorize species as either hunters of benthic invertebrates, facultative corallivores that perform both hunting and coral feeding, obligate corallivores, or zooplanktivores that feed selectively on zooplankton (Supplementary Table S1). Cole and Pratchett (2013) list *Heniochus acuminatus* as an invertivore based on anecdotal evidence from Michael (2004); however, we categorized this species as a selective zooplanktivore based on data from the IUCN and FishBase (Rocha et al. 2010; Froese and Pauly 2017).

We quantified the morphological diversity of butterflyfishes by building a dataset of eight traits, four of which are involved in the detection and capture of prey (feeding traits), and four are relevant to swimming performance (locomotor traits; Fig. 2 and Table 1). Morphological adaptations that increase the acceleration-based suction force exerted on prey are thought to enable the capture of evasive zooplankters. Forces exerted on prey may be increased by: (1) protruding the jaws further toward prey (Holzman et al. 2008; Staab et al. 2012); (2) decreasing mouth size (Wainwright et al. 2007); and (3) increasing the flow rate by either changing the absolute volume of the buccal cavity, decreasing the time it takes for contraction and expansion (Motta 1982), or increasing the force that initiates buccal expansion (Carroll et al. 2004). Feeding traits, therefore, included eye diameter, length of the ascending premaxillary process, width of the mouth at rest, and width of the head at the joint between the supracleithrum and post-temporal bone (S-PT joint; an indicator of the cross-sectional area of the epaxial muscles that produce force to initiate buccal expansion). Locomotor traits included body depth, maximum body width, caudal fin shape (described by the ratio of the inner length of the caudal fin to the outer length), and maximum body size measured as total length, compiled from FishBase (Froese and Pauly 2017). We measured 241 whole, cleared, and stained specimens (average number of specimens per species = 3; range = [1, 6]), representing 79 chaetodontid species (59% of nominal species; Fricke et al. 2020). Our sampling included 7 zooplanktivores (63.6% of nominal zooplanktivores; Cole and Pratchett 2013; Froese and Pauly 2017; Fricke et al. 2020; IUCN 2020); 8 Atlantic species (10.1%); and 71 Indo-Pacific species (89.9%; Supplementary Table S1)—reflecting the distribution of all nominal species between the major ocean basins (10.4% Atlantic and 89.6% Indo-Pacific).

We performed phylogenetically-informed analyses in the R statistical computing environment (R Core Development Team 2020) using the Bayesian posterior distribution of trees from Hodge et al. (2014). These phylogenetic trees were reconstructed from molecular data (mitochondrial 16S rRNA, 12S rRNA, *CO1*, cytochrome *b*; nuclear *TMO-4C4*, *S7* intron 1) and time-calibrated using fossil data. We sampled a total of 101 tree topologies, comprised of the maximum-clade credibility (MCC) tree, plus a random sample of 100 trees from the Bayesian posterior distribution, and trimmed them to match the species in our dataset. All morphometric measurements were aggregated as species’ means (except maximum body size, which was the maximum size reported, not a mean of multiple samples) and log_10_-transformed (except species’ mean caudal fin shapes, which were quantified as untransformed unit-less ratios). We accounted for variation in body size by regressing each morphometric trait (except maximum body size) on standard length using the phyl.resid function in phytools (Revell 2012) and the MCC tree (see Supplementary Table S2 for regression statistics). We also evaluated whether species with different foraging modes differed in the scaling of each morphometric trait with body size using the pgls function in caper (Orme et al. 2018). Further analyses were based on the phylogenetic residuals of the seven morphometric traits, and log_10_-transformed maximum body size.

### The evolutionary history of zooplanktivory

We inferred the evolutionary history of foraging mode for all 97 butterflyfish species present in the phylogenetic tree (Supplementary Table S1) with Bayesian stochastic character mapping in SIMMAP version 1.5 (Bollback 2006). Character histories were simulated on 101 trees with an empirical prior on the bias parameter, a branch length prior on the rate parameter, and the branches rescaled. Five single draws from the prior distributions were made for each tree, resulting in 505 stochastic character maps. Ancestral states were reconstructed on the MCC tree to explore transitions in foraging mode, and the relative amount of time spent in each character state.

### Effects of transitions to zooplanktivory on butterflyfish morphology

We compared a series of Brownian motion (BM) and Ornstein–Uhlenbeck (OU) models of trait evolution to determine the relative likelihood and location of morphological optima associated with foraging mode. BM models have a single parameter, *σ*^2^ that represents the rate of phenotypic change (Felsenstein 1985). In the single-rate BM (BM1) model, *σ*^2^ is constant and the continuous trait evolves according to a suite of random steps with no preferred direction. OU models are an extension of BM models, whereby a continuous trait evolves toward an optimal value (*θ*), with a constant strength of selection (*α*) and a constant rate of stochastic evolution around the optimum (*σ*^2^) (Hansen 1997; Beaulieu et al. 2012).

For each morphological trait, we used the R package OUwie (Beaulieu et al. 2012) to compare five different BM and OU models (BM1, OU1, BMS, OUM, and OUMV), in which up to two of the three parameters are allowed to differ along the phylogeny based on the inferred foraging regime at each node. Both the BM1 and the OU1 models allow morphological traits to evolve independently of foraging mode. The multi-peak OU models (OUM and OUMV) allow morphological traits to assume separate foraging dependent optima, while both the multi-rate BM (BMS) and the multi-peak, multi-rate OU (OUMV) models allow morphological traits to assume separate foraging dependent rates of trait evolution. If transitions to zooplanktivory have influenced the evolution of butterflyfish morphology, models that include multiple foraging dependent optima (OUM or OUMV) or multiple foraging dependent rates of trait evolution (BMS or OUMV) should be favored.

To account for uncertainty in phylogenetic and foraging mode reconstructions we fit evolutionary models across the set of 505 stochastic character maps, trimmed to match the species in our morphometric dataset. Morphological optima are estimated relative to the allometric expectation for all traits (except maximum body size), where positive values indicate traits larger than expected given body size, and negative values indicate traits smaller than expected given body size. After preliminary analyses, we found that dropping the estimated root parameter (*θ*_0_) from the models (i.e., setting root.station = TRUE) helped to stabilize parameter estimates. Therefore, we assumed that trait values at the root were within the distribution of the ancestral foraging regime (in this case, benthic invertivory for all analyses). We assessed model performance by examining the eigen-decomposition of the Hessian matrix and excluded iterations (model results and trees) with negative eigenvalues from the assessment of model fit and model averaging (see below).

For each trait, we assessed the goodness of fit of each model using Akaike weights derived from the size-corrected Akaike information criterion (AICc; Burnham and Anderson 2002; Burnham et al. 2011). Specifically, we calculated ΔAICc values for each retained iteration, averaged across iterations for each model, and used the mean ΔAICc values to calculate AICc weights. AICc weights describe the proportion of support a model receives in relation to support for all models (Burnham and Anderson 2002). Because AICc weights did not indicate strong support in favor of one particular model for most traits, we applied model-averaging to calculate reconstructed model parameters and their model-derived standard errors. To obtain the 95% confidence set of models, we ordered and then summed AICc weights from largest to smallest until the sum was just ≥0.95 (Burnham and Anderson 2002). Then, we recalculated AICc weights for those models retained in the 95% confidence set and used them to calculate average parameter values (*θ*, *α*, and *σ*^2^) and unconditional standard errors using the formulae provided in Burnham and Anderson (2002).

To visualize whether zooplanktivorous butterflyfishes are evolving toward morphological optima distinct from their benthivorous congeners, and for which traits, we plotted the evolutionary history of foraging mode as phenograms with the relative frequency of model-averaged optima at the tips. Traits evolving toward distinct, zooplanktivore-specific optima would have optimal distributions that overlap minimally with those of benthivorous regimes. Conversely, if the optimal distributions of all foraging regimes largely overlap, it would indicate that although zooplanktivores may be evolving toward a morphological optimum, it is not distinct from the optima estimated for benthivorous butterflyfishes.

Due to the inter-relatedness of the *α* and *σ*^2^ parameters in OU models, we interpret them together as the stationary variance (*σ*^2^/2*α*)—an estimate of the relative contribution of stochastic drift versus constraint (Butler and King 2004; Ho and Ané 2013). Lower stationary variance values indicate a greater influence of the constraining parameter (*α*) relative to stochastic drift (*σ*^2^), implying stronger attraction to the trait optimum. Thus, the evolution of a given morphological trait may be interpreted as more conserved for a foraging regime with a low stationary variance relative to other foraging regimes with higher stationary variances. Conversely, higher stationary variance values indicate a greater influence of σ^2^ rather than α, implying greater lability in the trait.

To visualize whether zooplanktivorous butterflyfishes have explored novel regions of morphospace we performed a phylogenetic principal component analysis (PCA) of all seven size-corrected morphological traits and maximum body size using phytools (Revell 2012). We specified the correlation matrix and obtained the correlation structure using maximum likelihood to optimize Pagel’s *λ*. We assessed the contribution of size to the first three PC axes by performing a phylogenetic generalized least squares regression on standard length using caper (Orme et al. 2018) and maximum likelihood to optimize Pagel’s *λ*. Then, we fit convex hulls to the PC scores of species in each foraging regime using the *chull* function (Eddy 1977). To estimate the location of adaptive optima on a multivariate adaptive landscape (Butler and King 2004) we performed the model-fitting methods described above on the first three principle component axes. We acknowledge that the use of phylogenetic PC axes as trait data can lead to biased results when the true model of trait evolution deviates from the model used to calculate the phylogenetic PCA axes, and that we are examining a biased sample of multivariate space by only considering the first three PC axes (Uyeda et al. 2015). We present this analysis only as a means to visualize the multi-dimensional trait space and the location of the estimated adaptive optima of zooplanktivores relative to benthivorous species; it is not used to assess convergence.

### The significance of convergence toward a zooplanktivorous ecomorph

We used the metrics implemented in the R package convevol (Stayton 2015) to measure the significance of convergence toward a zooplanktivorous ecomorph. We chose this method because it quantifies evolved similarity independently of evolutionary time and the distinctiveness of convergent phenotypes, both of which we consider separately in the analysis of individual traits. Therefore, if zooplanktivorous butterflyfishes have converged on a multi-dimensional morphological optimum indistinct from all forms of benthivory, this method has the power to detect it. Specifically, we quantified the degree to which zooplanktivorous lineages have evolved to be more similar using four distance-based measures (C_1_–C_4_) that calculate pair-wise distances between two lineages relative to the distance at the point in their evolutionary history when they were most dissimilar. C_1_ describes the proportion of the maximum distance between zooplanktivorous butterflyfish species that has been reduced by subsequent evolution (i.e., the relative amount of convergence). C_2_ describes the same distance in absolute terms (i.e., the maximum distance in morphospace between the lineages that unite zooplanktivorous species minus the distance between zooplanktivorous tips). C_3_ describes the proportion of total evolution attributable to convergence along lineages from the most recent common ancestor (MRCA) to extant zooplanktivorous species; and C_4_ describes the proportion of all of the evolution attributable to convergence in the smallest clade containing the zooplanktivorous species.

These metrics measure phenotypic distances based on ancestral state reconstruction (ASR) under a BM model. Because our model-fitting results suggest that most of the traits in our dataset are better fit by an OU model of trait evolution, and different underlying evolutionary models can alter ASR estimates and the resultant C metrics (Mahler et al. 2017), we also calculated each C-metric using ancestral states reconstructed under a multivariate OU model in Rphylopars (Goolsby et al. 2017). To test the significance of the observed C metrics we simulated trait evolution along the phylogeny according to a BM1 model 500 times using the variance-covariance matrix derived from the observed data and the MCC tree. We calculated convergence metrics for each simulated dataset and determined the proportion that was greater than the observed metrics (*P*-value). Finally, we repeated this analysis for each benthivorous group to determine whether they show significant ecomorphological convergence.

## Results

### The evolutionary history of zooplanktivory

**Fig. 3:**
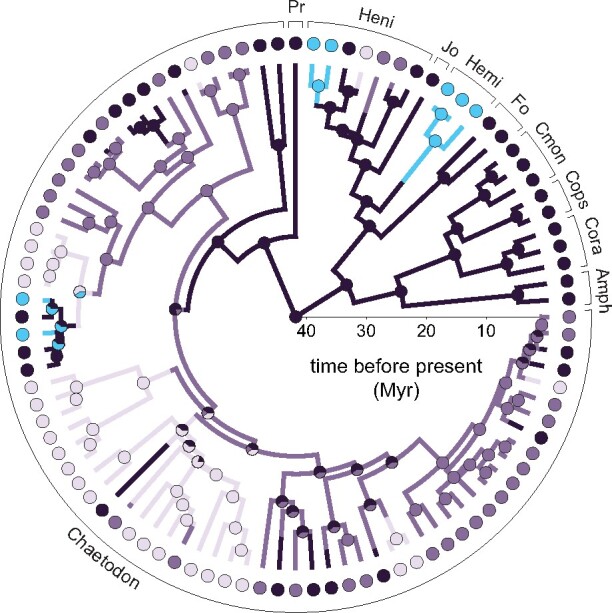
Evolutionary history of zooplanktivory (blue) and benthivory in butterflyfishes (benthic invertivores, dark purple; facultative corallivores, mid purple; obligate corallivores, light purple). A sample stochastic character map is shown on the MCC topology with posterior probabilities from five SIMMAPs summarized by pie charts at the nodes. Genera abbreviations include Pr = *Prognathodes*; Heni = *Heniochus*; Jo = *Johnrandallia*; Hemi = *Hemitaurichthys*; Cmon = *Chelmon*; Cops = *Chelmonops*; Cora = *Coradion*; and Amph = *Amphichaetodon*.

We found evidence for at least three relatively recent (within the past ∼19 million years) independent transitions to selective zooplanktivory within butterflyfishes, spanning three genera: *Hemitaurichthys*, *Heniochus*, and *Chaetodon* (Fig. 3 and Supplementary Fig. S1). Two transitions to zooplanktivory occurred from an ancestral state of benthic invertivory, one leading to the clade comprised of *H.acuminatus* and *Heniochus diphreutes*, and the other leading to the clade comprised of *Hemitaurichthys* spp., all of which are presently distributed throughout the Indo-Pacific (Supplementary Table S1). Within *Chaetodon*, transitions to zooplanktivory have arisen on the lineages leading to *Chaetodon guentheri* and *C. miliaris*; however, it is unclear whether these transitions occurred from ancestral states of benthic invertivory or corallivory. *Chaetodonguentheri* is presently distributed throughout the Central Indo-Pacific and Central Pacific regions, while *C. miliaris* is restricted to reefs around the Hawaiian Islands (Supplementary Table S1).

Across all stochastic character maps, we observed, on average, 28 transitions between foraging modes (Supplementary Table S3). Of these, 12.1% were transitions to zooplanktivory, 4.3% were reversals from zooplanktivory to benthic invertivory, and 1.4% were transitions from zooplanktivory to obligate corallivory. ASRs did not include transitions from zooplanktivory to facultative corallivory. Since they shared a common ancestor (∼42 Ma), butterflyfish lineages have cumulatively spent ∼4.4% of their evolutionary history with a zooplanktivorous foraging mode. The MRCA of all zooplanktivorous butterflyfishes corresponds to the MRCA of the family.

### Effects of transitions to zooplanktivory on butterflyfish morphology

Six of the seven morphometric traits, including ascending process length, body depth, body width, caudal fin shape, head width, and mouth width, increased with body size at rates that did not differ significantly between foraging modes (Supplementary Table S4). Eye diameter was the only trait to return a significantly different scaling relationship, whereby eye diameter increases more slowly with increasing body size among obligate corallivores relative to benthic invertivores (df = 4,74; *t* = −2.73; *P* < 0.05; Supplementary Table S4).

**Fig. 4:**
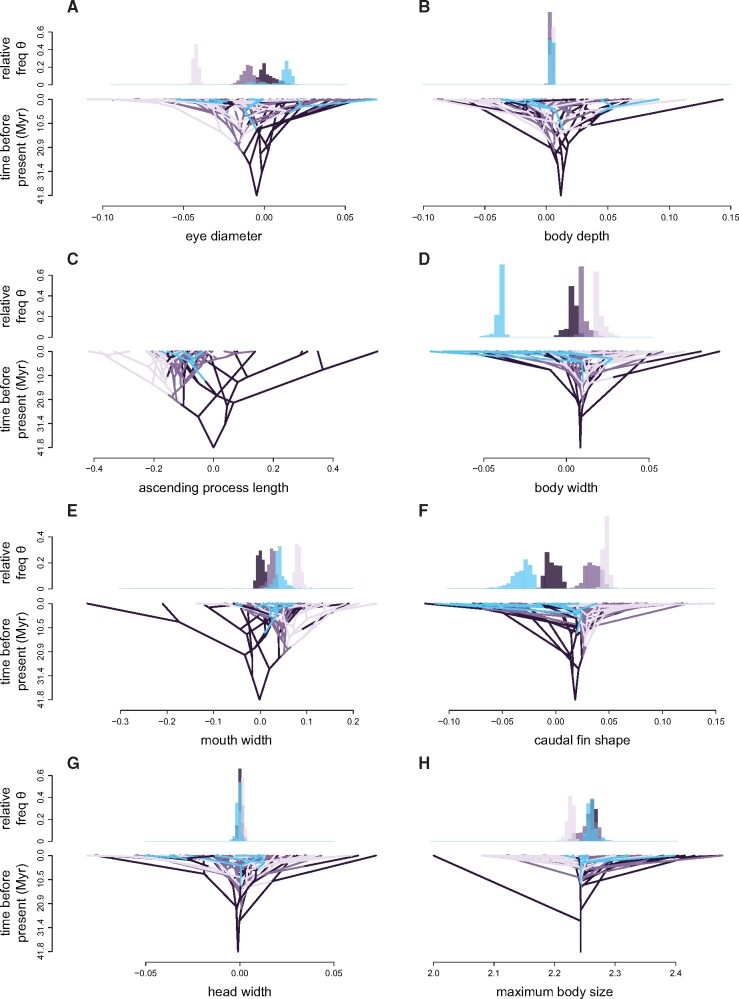
The MCC topology projected in a space defined by each of the (**A**, **C**, **E**, and **G**) feeding and (**B**, **D**, **F**, and **H**) locomotor traits (*x*-axes) and time (*y*-axes), with branches colored according to a sample stochastic character map (planktivores, blue; benthic invertivores, dark purple; facultative corallivores, mid purple; obligate corallivores, light purple). Ancestral states were estimated under a multivariate OU model for all traits except (C) ascending process length, which used a BM model. Above each phenogram [except (C)], histograms show the relative frequency of model-averaged optima, estimated from evolutionary model-fitting.

The 95% confidence set of models for eye diameter and body width contained only multipeak OU models (OUM and OUMV; Table 2), indicating these traits are evolving toward optima specific to each foraging mode. The remaining traits also included OU1 in model-averaging, which was favored over the multi-peak OU models for head width, body depth, and maximum body size, supporting a single, directional trend in the evolution of these traits, independent of foraging mode. The 95% confidence set of models for ascending process length included all models except OUMV, with BM1 receiving just over half of the total support (Table 2). Because BM and OU models differ in the parameters that determine trait variance (*σ*^2^ for BM versus *α* and *σ*^2^ for OU), and because the *θ* parameter in BM models represents the phylogenetic mean rather than the optimal value, we did not perform model averaging for ascending process length. Despite having the highest variance among the traits in our dataset, ascending process length was also the most phylogenetically conserved (*λ* = 1.01, *P* < 0.001). Most species’ ascending process lengths cluster near the estimated ancestral value, regardless of foraging mode; while species with extreme values, although noticeably distinct in foraging mode, are all closely related (Fig. 4C). It is clear that species with the longest ascending processes (*Forcipiger longirostris* and *Forcipiger flavissimus*) are exclusively benthic invertivores and species with the shortest ascending processes (*Chaetodon trifasciatus* and *Chaetodon lunulatus*) are exclusively obligate corallivores. However, the limited number of independent origins of these patterns within butterflyfishes likely precludes models favoring the adaptive evolution of ascending process length.

**Table 2:** Evolutionary models fit to morphological traits

	Number of retained iterations	Models	ΔAICc	AICc weight	Adjusted AICc weight
Feeding traits					
Eye diameter	503	OUM	0	0.940	0.952
		OUMV	5.955	0.048	0.048
Ascending process length	488	BM1	0.422	0.534	–
		OU1	2.183	0.221	–
		OUM	2.669	0.174	–
		BMS	5.686	0.038	–
Head width	502	OU1	0.002	0.888	0.908
		OUM	4.584	0.090	0.092
Mouth width	502	OUMV	1.159	0.548	0.548
		OU1	2.650	0.260	0.260
		OUM	3.257	0.192	0.192
Locomotor traits					
Body depth	494	OU1	0.010	0.903	0.937
		OUM	5.407	0.061	0.063
Body width	408	OUM	0	0.920	0.948
		OUMV	5.819	0.050	0.052
Caudal fin shape	499	OUM	0.148	0.793	0.793
		OU1	3.679	0.136	0.136
		OUMV	4.969	0.071	0.071
Maximum body size	505	OU1	0.250	0.623	0.623
		OUM	2.383	0.214	0.214
		OUMV	2.938	0.162	0.163
Phylogenetic PC axes					
PC1	497	OUMV	1.471	0.356	0.356
		OUM	1.670	0.322	0.322
		OU1	1.673	0.322	0.322
PC2	501	OUM	0.002	0.925	0.935
		OUMV	5.331	0.064	0.065
PC3	495	OUMV	0.374	0.791	0.821
		OU1	3.418	0.173	0.179

ΔAICc values, AICc weights, and adjusted AICc weights are provided for models retained in the 95% confidence set, with the exception of ascending process length, for which all model results are provided. Models are listed in decreasing order of AICc weight.

Model-averaged optima were distributed well within the range of observed trait values, indicating potential for species to reach the adaptive peaks associated with their respective foraging modes (Fig. 4). Optimal trait estimates for zooplanktivores were either indistinguishable from (head width, body depth, and maximum body size), intermediate to (mouth width) or distinct from (eye diameter, body width, and caudal fin shape) benthivorous optima (Fig. 4A–H). Eye diameter was the only trophic trait for which a distinct zooplanktivore-specific optimum was estimated (Fig 4A)—suggesting optimal eye size is larger in zooplanktivores relative to the optima estimated for benthivorous species. Still, other feeding traits not considered herein, such as dentition or gill raker length and spacing may be evolving toward novel zooplanktivore-specific optima. Of the locomotor traits, body width and caudal fin shape returned distinct zooplanktivore optima (Fig. 4D and F). Zooplanktivorous butterflyfishes have slimmer optimal body widths and more emarginate (curved inward, or concave) optimal caudal fin shapes compared with their benthivorous congeners (Fig. 4D and F and Supplementary Fig. S2). Estimates of the stationary variance reveal greater influence of the constraining parameter (*α*) relative to stochastic drift (*σ*^2^) in the evolution of maximum body size among zooplanktivores relative to benthivorous butterflyfishes (Supplementary Fig. S3E). A similar pattern was observed for the evolution of mouth width (Supplementary Fig. S3B). Despite separate foraging-dependent estimates of *σ*^2^ for eye diameter, body width, and caudal fin shape, the interplay between stochastic drift and constraint is comparable across foraging modes for these traits (Supplementary Fig. S3A, C, and D).

**Fig. 5:**
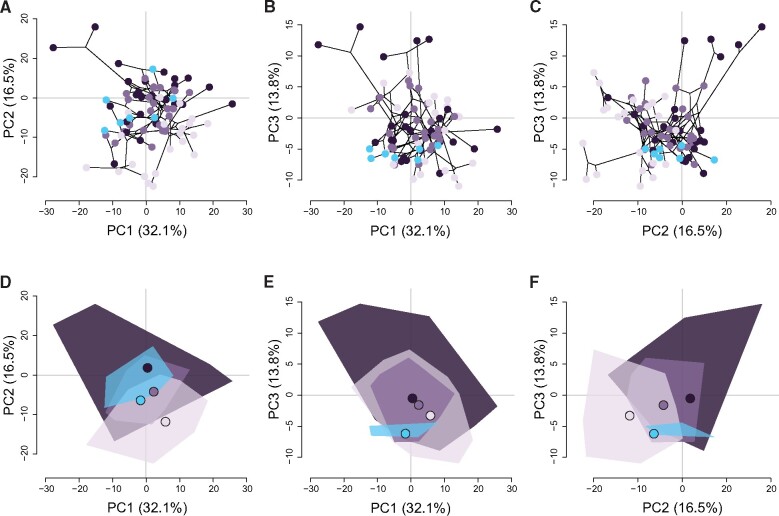
Phylomorphospace of planktivorous (blue) and benthivorous (benthic invertivores, dark purple; facultative corallivores, mid purple; obligate corallivores, light purple) butterflyfish species generated from a phylogenetic PCA of eight morphological traits. (**A**–**C**) The phylogeny is projected onto species’ PC scores along the first three PC axes. Note that ancestral states were estimated assuming a BM model of trait evolution. (**D**–**F**) Convex hulls indicate the areas of morphospace occupied by species with different foraging modes and their associated multivariate optima (large circles).

The first three axes of the phylogenetic PCA explain 62.4% of the morphological variation (Fig. 5) and are not significantly correlated with specimen length (Supplementary Table S5). PC1 primarily describes head, body, and mouth width, and body depth, while PC2 contrasts eye diameter, ascending process length, and maximum body size with caudal fin shape, and PC3 contrasts ascending process length and caudal fin shape with mouth width and maximum body size (Supplementary Fig. S4). Butterflyfishes with different foraging modes broadly overlap in the phylomorphospace, with some peripheral areas of the space occupied only by species with a particular foraging mode (Fig. 5). Zooplanktivorous butterflyfishes, notably *C. miliaris*, *C. guentheri*, *H. acuminatus*, and *Hemitaurichthysthompsoni*, have explored some novel areas of morphospace defined by a combination of the first three PC axes. Multivariate evolutionary optima suggest that zooplanktivores may be evolving toward slenderer body and head dimensions and more emarginate caudal fin shapes (Fig. 5 and Supplementary Fig. S2). Zooplanktivorous species lie fairly close to their optima on the first three PC axes, while benthivorous species are more widely dispersed around their respective optima. Although, model-fitting estimated a low evolutionary optimum along PC2, somewhat removed from the PC2 scores of three planktivores: *H.thompsoni* and both *Heniochus* species, suggesting that morphological convergence may be incomplete. Model-averaged stationary variance estimates suggest that the evolution of these linear combinations of traits may be more constrained in zooplanktivores relative to benthic invertivores whose trait evolution has been more labile (Supplementary Fig. S3F–H).

### The significance of convergence toward a zooplanktivorous ecomorph

Convergent evolution has reduced the morphological differences between zooplanktivorous butterflyfishes by 18.5–20% relative to the maximal spread of their ancestors. This proportion is significantly greater than expected under BM when ancestral states are reconstructed under BM (*P* < 0.05; Table 3); however, it becomes marginal when ancestral states are reconstructed under an OU process (*P* = 0.0599; Table 3). These values do not take into account the magnitude of change, as proportions apply equally to small or large distances in morphospace. Accounting for the absolute amount of evolution that occurred during convergence, zooplanktivorous butterflyfishes border on being significantly closer in morphospace than would be expected under BM (C_2_: 0.053–0.044; *P* = 0.05–0.11; Table 3). Therefore, while zooplanktivorous species are not entirely distinct from their benthivorous congeners, convergent evolution has increased morphological similarity among them. Convergence accounts for 6.6–7% of the morphological evolution along the lineages leading from the MRCA of zooplanktivorous species to the tips (*P* = 0.162–0.140; Table 3), and 0.3–0.5% of all of the evolution in the family (i.e., the smallest clade containing all zooplanktivorous species; *P* = 0.214–0.471; Table 3). To significantly exceed patterns of evolution expected under BM, these proportions would need to increase by less than 3% (2.6% and 0.31%, respectively, according to the BM ASR reconstructions; 2.13% and 0.47%, respectively, according to the OU ASR reconstructions).

**Table 3:** convevol results with ancestral states reconstructed (ASR) under a BM model and a multivariate OU model

			BM ASR		OU ASR	
		Cut-off	Observed	*P-*values	Observed	*P-*values
C_1_	bi	0.1332	0.0936	0.5110	0.0817	0.7685
	fc	0.1417	0.1065	0.3473	0.0800	0.7884
	oc	0.1379	0.0837	0.6487	0.0868	0.5948
	zp	0.1978	0.2012	0.0479*	0.1853	0.0599
C_2_	bi	0.0392	0.0306	0.1936	0.0259	0.3872
	fc	0.0336	0.0258	0.2136	0.0163	0.7525
	oc	0.0303	0.0233	0.2056	0.0216	0.2954
	zp	0.0539	0.0532	0.0539	0.0435	0.1078
C_3_	bi	0.0646	0.0399	0.7405	0.0378	0.8064
	fc	0.0667	0.0417	0.6727	0.0346	0.8822
	oc	0.0675	0.0377	0.7585	0.0403	0.6647
	zp	0.0911	0.0655	0.1617	0.0698	0.1397
C_4_	bi	0.0053	0.0027	0.8204	0.0018	0.9900
	fc	0.0046	0.0023	0.7924	0.0011	0.9980
	oc	0.0042	0.0020	0.7944	0.0015	0.9661
	zp	0.0077	0.0046	0.2136	0.0030	0.4711

Regimes are denoted as (bi) benthic invertivores, (fc) facultative corallivores, (oc) obligate corallivores, and (zp) selective zooplanktivores. Significant values are denoted with an asterisk.

## Discussion

Selective zooplanktivory is considered a derived foraging mode among acanthopterygians whose early ancestors were adapted to feed on relatively large prey (Gosline 1971; Kotrschal 1988). However, modern zooplanktivores evolved within lineages whose feeding morphologies were already modified for a range of tasks, as evidenced by their occurrence in many of the major coral reef fish families (Hobson 1991; Wainwright and Bellwood 2002). Transitions to selective zooplanktivory from different types of non-planktivorous foraging, considered with varying degrees of specificity, have produced inconsistent morphological changes (Wainwright et al. 2002, 2004; Cooper and Westneat 2009; Aguilar-Medrano et al. 2011; Schmitz and Wainwright 2011; Frédérich et al. 2013; Friedman et al. 2016; Cooper et al. 2017; Tavera et al. 2018), alluding to the potential for differences in ancestral condition to affect the evolutionary trajectory and magnitude of associated morphological change. Within butterflyfishes, a group with multiple modes of benthic foraging—all of which target small prey, we show that the functional demands of selective zooplanktivory are associated with novel morphological optima for a subset of traits related to prey detection and locomotion. Our results are consistent with the occurrence of evolutionary change in locomotory morphology between benthic and pelagic or limnetic habitats in other lineages (Schluter 1993; Wainwright et al. 2002; Hulsey et al. 2013; Kusche et al. 2014; Tavera et al. 2018; Friedman et al. 2020); however, the nuances of trait change are novel and further suggest that differences between these environments are independent of selective pressures related to prey size.

Ancestral lineages adapted to forage on small prey may not require drastic changes in feeding morphology upon transitions to selective zooplanktivory. Although some benthivorous butterflyfish species have highly specialized diets, the morphological adaptations that facilitate high precision feeding on small, benthic prey appear functionally versatile (Fig. 4A, C, E, and G). Such versatility has likely allowed benthivorous lineages to exploit similarly-sized prey in mid-water when such resources are abundant or when competition is reduced (Liem 1980; Robinson and Wilson 1998; Bellwood et al. 2006; Golcher-Benavides and Wagner 2019). Indeed, many butterflyfish species are opportunistic and will abandon their preferred prey to feed exclusively on abundant plankton for up to a few days (Motta 1988). Moreover, dietary flexibility has been documented among the more generalized coral-feeding butterflyfishes in response to prey availability (Pratchett et al. 2004; Berumen et al. 2005) or the presence of superior competitors (Cox 1994; Berumen and Pratchett 2006). Notably, 8 of the 11 zooplanktivorous butterflyfish species are primarily distributed outside of the hotspot of reef fish biodiversity (i.e., the majority of their distribution lies outside of the IAA; Fig. 1E). One such peripheral region, Hawai’i, is characterized by a relatively depauperate zooplanktivore community (Hourigan and Reese 1987), where *C.miliaris—*a zooplanktivore, is the most common butterflyfish species (Motta 1982). *Chaetodon kleinii*, a facultative corallivore throughout most of its range (Sano 1989; Pratchett 2005), is also known to feed on zooplankton in Hawaii (Hobson 1974). If competition for zooplankton were reduced in other peripheral regions, our results suggest that populations of benthivorous butterflyfishes would be readily able to exploit the resource with their existing trophic morphologies. This implies that reduced competition with other zooplanktivorous lineages may have played an important role in the replicated evolution of selective zooplanktivory among Indo-Pacific butterflyfishes.

There are only 14 butterflyfish species in the Atlantic; therefore, it is difficult to draw conclusions regarding the absence of zooplanktivores from this realm (Fig. 1E). Twelve of the Atlantic species are benthic invertivores (86%), and two are facultative corallivores (*Chaetodon capistratus* and *Chaetodon ocellatus*), pointing to a general lack of diet diversity relative to butterflyfish communities in the Indo-Pacific, which could influence the likelihood of transitions to zooplanktivory. However, we found that transitions to zooplanktivory occurred most frequently among lineages whose ancestral foraging mode was benthic invertivory (on average, 2.8 of the 3.4 transitions; see Supplementary Table S3), suggesting that low diet diversity among Atlantic butterflyfish species does not account for the paucity of zooplanktivores. Phylogenetic diversity of butterflyfishes has been shown to mirror alpha diversity, such that butterflyfish species within the Atlantic regions have low phylogenetic diversity and species share more evolutionary history than expected under a null model (Kulbicki et al. 2014). This could constrain the likelihood of transitions to zooplanktivory by limiting variation and increasing the influence of ancestral effects. However, these mechanisms are unlikely since two Atlantic species, *Chaetodon sanctaehelenae* and *Chaetodon sedentarius* form a clade and share a recent common ancestor (∼2.7 Ma) with the only two *Chaetodon* zooplanktivores, *C. miliaris* and *C. guentheri*. Through process of elimination, we speculate that differences in geological history and environmental factors likely underly the absence of zooplanktivorous butterflyfishes in the Atlantic, although more work should be done on this topic.

Zooplanktivore-specific optima estimated for traits related to prey detection and capture did not consistently support the predicted patterns relative to optima estimated for benthivorous foraging modes (Fig. 4A, E, and G), suggesting that butterflyfish foraging is at least partially modulated by behavior. On average, predators have larger eyes than browsers and grazers because higher acuity is required to detect and localize prey (Litherland and Collin 2008; Veilleux and Kirk 2014). The expectation that, as hunters of small prey, zooplanktivores have larger eyes relative to their non-zooplanktivorous counterparts has mixed support and may depend on the nature of the ancestral foraging mode (Schmitz and Wainwright 2011; Friedman et al. 2016). Our results support the predicted differences in eye size between hunters and browsers/grazers: with optimal eye size increasing as the amount and type of hunting described by each foraging mode increases (Fig. 4A). Zooplanktivores have an optimal eye size slightly larger than the allometric expectation and the optimum estimated for benthic invertivores. Benthic invertivores and zooplanktivores both hunt evasive prey, although the transparency of zooplankton against a low-contrast background may require higher visual acuity to detect compared with similarly-sized prey on the benthos (Jones 1968; Davis and Birdsong 1973; Lazzaro 1987; Fernald 1990; Wainwright and Richard 1995; Johnsen 2001; Wainwright and Bellwood 2002). Despite estimates of separate, foraging dependent optima, the variation in eye size around each optimum suggests that most benthivorous species could modulate their behavior and successfully detect planktonic prey.

Notably, obligate corallivores had the most distinct optimal eye diameter—smaller than all other optima and the allometric expectation. Moreover, the rate of increase in eye diameter with body size is significantly slower in obligate corallivores (Supplementary Table S4), which could reflect a trade-off or constructional constraints that limit eye size. For example, in cichlids and cyprinids, eye size is negatively correlated with the mass of the adductor mandibulae muscles (Barel 1982; Hulsey and Hollingsworth 2011). Interestingly, all obligate corallivores belong to the genus *Chaetodon*, which is united by flexion at the intramandibular lower jaw joint (IMJ), a joint between the dentary and articular that facilitates dorso-ventral rotation of the dentary tooth surfaces (Konow et al. 2008; Konow and Ferry-Graham 2013). The intramandibular jaw joint has evolved independently in several other groups of reef-associated benthivorous fishes, including, among others, parrotfishes (Family: Labridae), surgeonfishes (Family: Acanthuridae), and angelfishes (Family: Pomacanthidae), which articulate the IMJ via modifications of the adductor mandibulae musculature (Purcell and Bellwood 1993; Bellwood 1994; Streelman et al. 2002). Within butterflyfishes, flexion at the IMJ is reported to increase with increasing corallivory (Konow and Ferry-Graham 2013). Although our results would be consistent with a constructional constraint on eye size in taxa that possess an IMJ, confident determination of a negative relationship between the mass of the adductor mandibulae, or other muscles in the head and eye size requires further analysis of morphological evolution.

Many benthivorous fishes are able to feed in mid-water using suction rather than a combination of suction and biting (Liem 1980; Pratchett et al. 2001; Golcher-Benavides and Wagner 2019), and our results suggest most butterflyfishes are also capable of such behavioral modulation (Copus and Gibb 2013). Morphometric measurements of the three traits capable of increasing suction strength (ascending process length, mouth width, and head width) indicate that zooplanktivores likely produce suction strengths similar to many benthivorous species, such as *Chaetodon argentatus*, *Chaetodon vagabundus*, *Heniochus chrysostomus*, *Heniochus monoceros*, and *Coradion altivelis* (Fig. 4C, E, and G). While the length of the ascending premaxillary process is a direct indicator of the degree of jaw protrusibility in most teleost fishes (Liem 1970; Gosline 1981; Bellwood et al. 2015—see also Staab et al. 2012), the strength of this form–function relationship within butterflyfishes is unclear as at least one species, *F.longirostris*, achieves significantly greater protrusion than its congener, *F.flavissimus*, through the use of a novel joint (Motta 1984b; Ferry-Graham et al. 2001). Therefore, kinematic excursions quantified from live (sensu Ferry-Graham et al. 2001; Cooper et al. 2017) or manipulated specimens (sensu Motta 1988) are recommended to assess whether zooplanktivorous butterflyfishes are capable of greater jaw mobility despite their intermediate morphology. Estimates of optimal mouth width suggest that feeding obligately on non-evasive coral prey may relax the need to produce strong suction forces. Some amount of suction may help corallivores targeting individual coral polyps to bite them before they are retracted, but for other species, such as *C.trifasciatus*, a wider mouth facilitates the capture of multiple coral polyps per bite while combing the coral surface (Motta 1985, 1988). Estimates of optimal head width did not differentiate foraging modes and variance around the optima indicates that the muscle mass responsible for initiating suction in zooplanktivores is comparable to many benthivorous species. Overall, our results indicate that the suction forces generated during the capture of small benthic prey are strong enough to capture similarly sized planktonic prey in mid-water.

In contrast to feeding traits, we observed notable morphological specializations in locomotor traits following transitions to zooplanktivory (Fig. 4B, D, F, and H). Foraging on the benthos requires precise maneuvers, while foraging in mid-water typically involves less maneuvering and more efficient sustained swimming (Plaut 2001; Ohlberger et al. 2006). Our results show that while body depth is highly conserved among butterflyfish species (Fig. 4B), zooplanktivores evolved more narrow body widths (Fig. 4D)—a potentially important, but often over-looked axis of morphological diversity among teleostean fishes (Price et al. 2019). Changes in body width impact body fineness—calculated as standard length divided by the square root of maximum body depth times maximum body width—which is expected to negatively correlate with the magnitude of form drag (Bainbridge 1960; Blake 1983; Sfakiotakis et al. 1999). All else being equal, a reduction in butterflyfish body width increases their fineness ratio (average = 2.9) toward the value that maximizes volume with minimum surface area (optimum = 4.5; Blake 1983), thereby potentially reducing form drag. Moreover, hydromechanical interactions between the body and fins of median-paired fin (MPF) swimmers like butterflyfishes can produce energetic advantages including high hydromechanical efficiency, thrust enhancement, and drag reduction that overcome the kinematic and morphological constraints necessary for efficient propulsion of body-caudal fin swimmers (Blake 2004). Further work is needed to explore the role of body width in hydromechanical interactions and the metabolic cost of transport in MPF swimmers.

Differences in body shape have been associated with habitat use along the benthic to pelagic or limnetic axis in many other freshwater and marine fish lineages (Schluter 1993; Wainwright et al. 2002; Hulsey et al. 2013; Kusche et al. 2014; Tavera et al. 2018; Friedman et al. 2020), and one of the most common modifications associated with pelagic or limnetic habitat use is a reduction of body depth. Yet, zooplanktivorous butterflyfishes maintain a shape that is among the most-deep bodied of all fishes (Price et al. 2015; Hodge et al. 2018). Their commitment to an extreme, deep-bodied form may reflect developmental or other constraints that limit the potential for modification. Our results suggest that mid-water living imposes further constraints on the evolution of maximum body size (Supplementary Fig. S3E). Across marine teleosts, transitions to pelagic living were associated with increased body elongation, achieved via increases in standard length combined with reductions in body depth and width (Friedman et al. 2020). However, because both size and body depth are generally conserved across butterflyfishes, adaptation to mid-water foraging appears limited to modification of body width. These findings are consistent with the notion that when size is constrained or niche limited, adaptation requires modifications to morphological proportions (Zelditch et al. 2017).

The evolution of body width toward a novel, distinct zooplanktivore optimum over a rather short amount of evolutionary time was likely enabled by a uniformly high rate of morphological evolution, which was higher than any of the other trait-specific rate estimates (Fig. 4D and Supplementary Table S6). Relative to other foraging modes, zooplanktivores and benthic hunters also returned higher rates of caudal fin shape evolution with trajectories toward separate morphological optima (Fig. 4F and Supplementary Table S6). Moreover, we found that body width and caudal fin shape have evolved synergistically, such that as body widths narrowed, caudal fins became more emarginate (*F* = 4.88; df = 1.77; *P* = 0.030). This is consistent with the functional linkage hypothesis that predicts swimming efficiency can be maximized if the structures directly involved in propulsion exhibit correlated evolution toward forms that minimize the expenditure of energy (Feilich 2016). Species that specialize in steady swimming, such as zooplanktivores, are expected to have fusiform body shapes with forked caudal fins to reduce drag and increase thrust during body-caudal swimming (Table 1; Lighthill 1970; Webb 1984a; Weihs 1989; Feilich and Lauder 2015). Butterflyfish combine body-caudal swimming with pectoral fin rowing (MPF swimming; Webb 1984a; Gerstner 1999; Blake 2004), which is thought to facilitate maneuverability within close range of complex habitat topographies (Konow and Ferry-Graham 2013; Floeter et al. 2018). Indeed, MPF swimming has been shown to reduce costs associated with increasing frequency of direction changes (Marcoux and Korsmeyer 2019). Although our results do support the predicted morphological adaptations to mid-water swimming, we suspect that zooplanktivorous butterflyfishes have retained much of the maneuverability expressed by their benthivorous counterparts, as it may also be advantageous for selecting planktonic prey. Moreover, the evolutionary optima we describe do not necessarily translate directly to biomechanical optima, particularly in the absence of experimental work comparing locomotor performance among butterflyfish species. Future studies should evaluate the relative use of steady swimming and maneuverability among species with different foraging modes in laboratory and field settings to determine whether the morphological modifications we observed translate to functional differences.

Overall, the novel, distinct nature of several locomotor trait optima suggests that benthic and mid-water foraging impose divergent selective pressures capable of influencing body shape regardless of whether changes in feeding morphology also occur. This is consistent with the decoupling of diversification dynamics between head and body morphologies described more broadly across actinopterygians and supports the idea that evolutionary modularity enhances morphological diversity (Larouche et al. 2018). Nevertheless, feeding and locomotor traits do not operate in isolation, but rather as components of a system with many complex interactions. The foraging-specific adaptive optima estimated within our multivariate trait space suggest that zooplanktivores may be converging toward an ecomorphology, but that it is generally not distinct from the trait space occupied by benthivorous species (Fig. 5). Convergent patterns of multivariate trait evolution, specifically C_1_ and C_2_, straddle significance, demonstrating that convergent evolution has closed some of the morphological distance between zooplanktivorous species (Table 3). Given the relatively recent evolution of zooplanktivory, the inconsistency of morphological adaptations, and evidence of morphological constraint, it was not surprising that convergence does not account for a significant proportion of the evolution that has occurred since the origin of the family (C_3_ and C_4_; Table 3). Although none of the convergence metrics include a time component, because planktivory arose relatively recently among species that are distantly related (i.e., their MRCA is the root node of the phylogeny), there are long branches and many lineages considered in both the C_3_ and C_4_ metrics. It was, however, surprising that each metric would need to increase by less than 3% to significantly exceed patterns produced by BM.

Butterflyfish have a long history of feeding on small, benthic prey, which has undoubtedly impacted their morphology. Following transitions to selective zooplanktivory, morphological traits involved in the procurement of small prey remained largely comparable with benthivorous species, while key locomotor traits have adapted to the functional demands of mid-water foraging. Although zooplanktivorous butterflyfishes do not generally exhibit fast rates of morphological evolution relative to benthivorous species (Supplementary Table S6 and Supplementary Fig. S3), they achieved nearly significant convergence over a period of time that cumulatively accounts for ∼4.4% of the evolutionary history of the family (Table 3 and Fig. 3). This stands in contrast to all of the benthivorous butterflyfish lineages that have had much more time to accumulate morphological changes (Fig. 2), but none of which have significantly converged on an ecomorphology described by this set of feeding and locomotor traits (Table 3). Taken together, these findings indicate that functional demands associated with zooplanktivory are capable of imposing strong selective pressures on morphology. Our results are consistent with the notion that when body size is constrained, adaptation of shape may result in convergence when functions are challenging (Zelditch et al. 2017).

The method we used to quantify the significance of convergence does not account for the distinctiveness of convergent phenotypes (Stayton 2015) and we specifically chose it for that reason. We suggest that this type of indistinct convergence can reveal the relative roles of adaptation and exaptation in shaping convergent patterns. Whether transitions to selective zooplanktivory in other groups have resulted in widespread morphological convergence, and whether convergence involves traits that function in the detection and capture of prey or swimming performance will likely depend on the ancestral foraging regime. For example, would we see similar trait changes in groups where ancestral foraging took place in mid-water? Investigating this across a broad taxonomic scale where transitions occur from disparate ancestral selective regimes would provide more insight (sensu Moen et al. 2016). The discretization of variation among non-focal taxa will obviously influence the distinctiveness of focal taxa. For example, had we considered all benthivorous foraging modes as non-planktivorous, we likely would not have recovered distinct peaks for morphological traits that returned intermediate zooplanktivore optima. Although the convergence analyses consider only focal and non-focal taxa, a more fine-scale representation of the diversity of benthivorous and non-planktivorous foraging modes in complementary analyses has the power to reveal important morphological modifications associated with transitions to different forms of planktivory, or other mid-water foraging modes.

## Authors’ contributions

J.R.H. conceived the study and developed the study design with input from P.C.W. and S.A.P. J.R.H., Y.S., M.A.W., A.M., B.T., A.Š., and A.S.R. prepared, cleared, and stained specimens. J.R.H., Y.S., M.A.W., A.M., and A.Š. collected the data. J.R.H. analyzed the data with input from S.A.P. J.R.H. wrote the manuscript with contributions from all authors.

## Supplementary Material

obab014_Supplementary_Data
